# Design of 400 V Miniature DC Solid State Circuit Breaker with SiC MOSFET

**DOI:** 10.3390/mi10050314

**Published:** 2019-05-10

**Authors:** Hui Li, Renze Yu, Yi Zhong, Ran Yao, Xinglin Liao, Xianping Chen

**Affiliations:** 1 State Key Laboratory of Power Transmission Equipment & System Security and New Technology, School of Electrical Engineering, Chongqing University, Chongqing 400044, China; yurenze1996@126.com (R.Y.); yizhong1994@outlook.com (Y.Z.); yaoran1234@163.com (R.Y.); 2 Energy Internet Engineering Technology Center of Chongqing City, Chongqing University of Technology, Chongqing 400054, China; lxl108381@126.com; 3 Key Laboratory of Optoelectronic Technology & Systems, Education Ministry of China, and College of Optoelectronic Engineering, Chongqing University, Chongqing 400044, China

**Keywords:** silicon carbide (SiC) metal-oxide-semiconductor field-effect transistors (MOSFETs), solid state circuit breaker (SSCB), prototype, circuit design

## Abstract

Silicon carbide (SiC) metal-oxide-semiconductor field-effect transistors (MOSFETs) have the advantages of high-frequency switching capability and the capability to withstand high temperatures, which are suitable for switching devices in a direct current (DC) solid state circuit breaker (SSCB). To guarantee fast and reliable action of a 400 V DC SSCB with SiC MOSFET, circuit design and prototype development were carried out. Taking 400V DC microgrid as research background, firstly, the topology of DC SSCB with SiC MOSFET was introduced. Then, the drive circuit of SiC MOSFET, fault detection circuit, energy absorption circuit, and snubber circuit of the SSCB were designed and analyzed. Lastly, a prototype of the DC SSCB with SiC MOSFET was developed, tested, and compared with the SSCB with Silicon (Si) insulated gate bipolar transistor (IGBT). Experimental results show that the designed circuits of SSCB with SiC MOSFET are valid. Also, the developed miniature DC SSCB with the SiC MOSFET exhibits faster reaction to the fault and can reduce short circuit time and fault current in contrast with the SSCB with Si IGBT. Hence, the proposed SSCB can better meet the requirements of DC microgrid protection.

## 1. Introduction

Direct current (DC) microgrids have elicited increasing attention in recent years, because they have a simple structure and are easy to control [1,2]. However, the safe and stable operation of a DC microgrid is inseparable from the effective protection technology [3]. When a short circuit occurs, fault current will rise instantly to a considerable extent. The equipment in the power system will suffer from huge electro-thermal stress, which seriously affects the operational reliability of the system [4,5]. Therefore, a circuit breaker is required to isolate the fault. Existing circuit breaker techniques can be classified into three types: mechanical circuit breaker, hybrid circuit breaker, and solid state circuit breaker [6,7,8,9,10]. The function of a mechanical circuit breaker is achieved by a mechanical switch. Although the mechanical circuit breaker can handle high current, electric arcing caused contact erosion reduces the lifetime and the disadvantage of long break time further limits its application. A hybrid circuit breaker is composed of a mechanical switch and parallel power devices. The current flows into the mechanical switch under normal working condition and transfers into the semiconductor switch under fault condition. The loss of a hybrid circuit breaker is minimal, but the control of a hybrid circuit breaker is complex. A solid state circuit breaker (SSCB) is composed of power devices and related circuits to realize the interruption of fault current. The fault clearing time of an SSCB is short, but the drawback of an SSCB lies in its loss. By comparison, an SSCB responds rapidly to the fault and produces no arc when cutting off the current. Besides, without the interaction of mechanical switch and power switch, the control of an SSCB is not complex and the reliability is relatively high. Therefore, an SSCB can better meet the fast and reliable protection requirements in DC microgrid, and has received extensive concern.

The commonly used devices in SSCBs are Silicon (Si)-based power devices [9,11,12,13,14]. However, the performance of traditional Si devices has reached its limitation [15]. With the improvement of wide-bandgap semiconductor technology and its application in SSCBs, the performance of SSCBs can be further improved. Compared with Si devices, wide-bandgap semiconductor Silicon Carbide (SiC) devices exhibit further excellent properties. The comparison of material properties is provided in Table 1 [16]. Wider bandgap and higher thermal conductivity indicate that SiC devices can withstand higher temperature than Si devices, which reduces heat dissipation requirement of an SSCB. Higher electron velocity guarantees faster switching speed, higher frequency characteristics, and higher current density of SiC-based power devices. The aforementioned advantages enable SiC devices to operate faster and withstand higher temperature than Si devices, which break through the limitation and improve the fault response capability of Si-based SSCB [17,18].

Among SiC power devices, SiC metal-oxide-semiconductor field-effect transistors (MOSFETs) exhibit the most promising prospects [19,20], but study on SSCBs with SiC MOSFET is still in its infancy. Zhou et al. [21] introduced a digital SSCB with SiC MOSFET to identify inrush current, but the fault clearing time was long, which is adverse to the equipment protection in the power system. Ren et al. [22] focused on solving the inconsistency voltage distribution on cascaded SiC MOSFETs in SSCB in high voltage direct current (HVDC) transmission application, while the detailed design of SSCB was not given. Zhang et al. [23] proposed a changeable delay time protection method for SSCB with SiC MOSFET. However, the high requirements to parameter calculation and complicated design make the SSCB hard to realize. Therefore, to achieve quick and reliable action of the SSCB and to make the SSCB easy to realize in application, detailed design of the SSCB with SiC MOSFET should be carried out.

In this study, a 400 V DC microgrid was adopted as the application background. The topology and structure of an SSCB with SiC MOSFET were introduced. The design of the SSCB was described in detail, including device selection, gate driver, fault detection circuit, energy absorption circuit, and snubber circuit. Finally, a prototype of the DC SSCB with SiC MOSFET was developed, tested and compared with Si insulated gate bipolar transistor (IGBT). Experiment results proved that the designed SSCB with SiC MOSFET can operate reliably in case of failure and can reduce voltage spike during the turn-off period. In addition, the designed SSCB exhibits fast interrupting characteristics, which provide guidance for improving the performance of SSCB and the application of SSCB with SiC MOSFET.

## 2. Topology of the SSCB with SiC MOSFET

**Figure 1:**
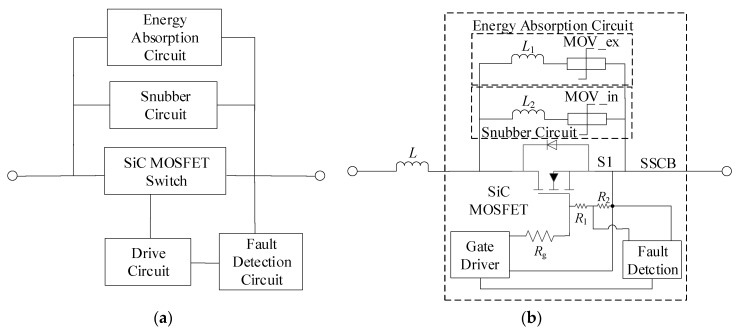
(**a**) Topology of the solid state circuit breaker (SSCB) with SiC metal-oxide-semiconductor field-effect transistor (MOSFET); (**b**) Structure of the SSCB with SiC MOSFET.

Figure 1a demonstrates the topology of the SSCB with SiC MOSFET. As illustrated in the figure, the SSCB with SiC MOSFET is composed of a SiC MOSFET switch, a drive circuit, a fault detection circuit, an energy absorption circuit, and a snubber circuit. The functions of the power device are to conduct current and to cut off the circuit when fault occurs. The drive circuit is used to send control signals to the power device to turn it on or off. The fault detection circuit can immediately detect the faults and react. The energy generated during a fault period is absorbed and dissipated by the energy absorption circuit. The snubber circuit is used to suppress the voltage spike induced by circuit inductance in the initial turn-off stage to protect the voltage on the device from exceeding the rated voltage. The structure of the SSCB is depicted in Figure 1b to specifically show its components and further explain the working principle of the SSCB. The gate driver is connected to the SiC MOSFET by gate resistance *R*_g_. The fault detection circuit detects variations in gate voltage. *R*_1_ and *R*_2_ are divider resistances. The function of energy absorption circuit is realized by using a metal-oxygen-varistor (MOV), and the snubber circuit is achieved by simply using MOV_in instead of the commonly used resistance-capacitor (RC) or resistance-capacitor-diode (RCD) circuit [24]. *L*_1_ and *L*_2_ are the parasitic inductances.

The working principle of the SSCB of SiC MOSFET can be explained as below: S1 conducts and current flows through SiC MOSFET under normal working condition. The resistance of MOV_in and MOV_ex are high, and no current flows into the snubber circuit and the energy absorption circuit. When abnormal state occurs, the fault current increases rapidly and causes the change of gate voltage. Through logical judgement and comparison, a turn-off signal is sent to gate driver to turn off the SiC MOSFET. Simultaneously, the voltage on SSCB reaches to the breakdown voltage of MOV, the resistance of MOV immediately drops to a very small value and the voltage on SSCB is clamped. First, MOV_in is triggered to operate to suppress the voltage spike in the early turn-off stage. Then, MOV_ex operates and because of the voltage difference between MOV_in and MOV_ex, the fault current transfers to energy absorption circuit. During this time, the voltage on MOV_ex is higher than the DC-link voltage. MOV_ex in absorbs the energy and fault current attenuates gradually. When the current reaches to zero, the SSCB stops working and fault is cleared.

## 3. Design of the SSCB with SiC MOSFET

### 3.1. Device Selection

The designed SSCB is used in a 400 V DC microgrid. A high induced voltage *V*_L_ is generated at the early turn-off period due to the fast switching speed and the existence of parasitic inductance in the circuit. Therefore, the SiC MOSFET is required to withstand the sum voltage of DC-link voltage *V*_DC_ and *V*_L_. *V*_L_ is determined by the parasitic parameters in the circuit and change rate of the current. In consideration of the magnitude of the inductance in circuit board (less than 0.2 μH) and the di/dt for SiC MOSFET in turn-off stage (1–3 A/ns) [25], the induced voltage *V*_L_ may reach 200 V or above. Therefore, the withstand voltage of the device is preferably guaranteed to have larger margin in application [21,26]. Considering the SSCB can be used under high current condition, devices with high rated current can be selected. To date, the major manufacturers of SiC MOSFETs are CREE (Durham, NC, USA) and ROHM (Kyoto, Japan). Both manufacturers have products with relatively high rated current: C2M0080120D of 36 A, SCT2080KE of 40 A. However, the device must have a stronger short circuit withstand capability to assure the reliability of the SSCB. According to the study of Wang et al. [27], the comparison of short circuit withstand time (SCWT) and critical energy of the 1200 V/80 mΩ CREE and ROHM SiC MOSFET are made in Table 2. The SCWT of ROHM SiC MOSFET is higher than CREE SiC MOSFET under same DC-link voltage or temperature. In addition, the critical energy of ROHM product is also greater than CREE product. Thus, the 1200 V/80 mΩ ROHM SiC MOSFET performs better than CREE SiC MOSFET in harsh condition. Accordingly, SCT2080KE, with the rated voltage of 1200 V and rated current of 40 A is selected as the switching device of the SSCB.

### 3.2. Drive Circuit

The suitable drive circuit is the prerequisite for ensuring the satisfactory operation of the SSCB with SiC MOSFET. To assure the fast switching speed, it is necessary to select the proper gate voltage. In addition, the design of the drive circuit must also consider the value of gate resistance, the selection of driver chip.

#### 3.2.1. Gate Voltage

**Figure 2:**
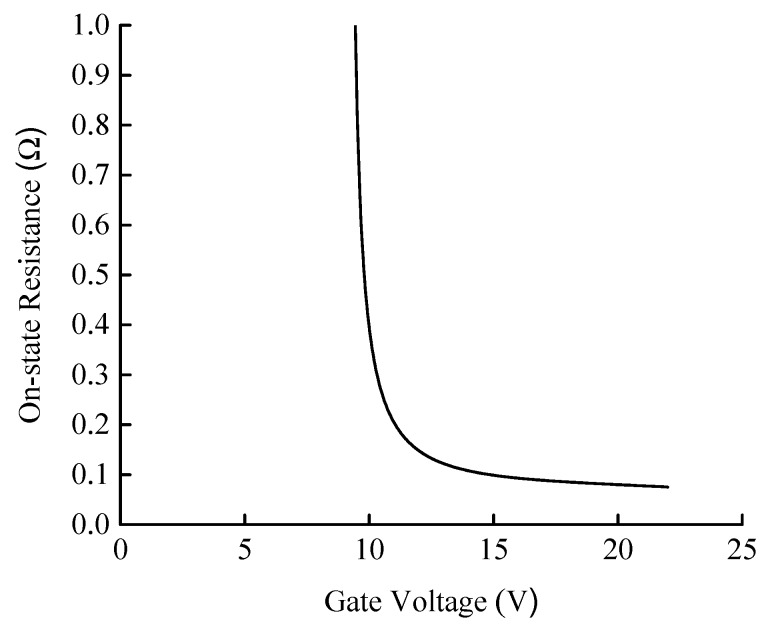
Relationship between gate voltage and on-state resistance of the SiC MOSFET.

Gate voltage is crucial to develop the excellent characteristics for the SiC MOSFET. Figure 2 shows the relationship between gate voltage and on-state resistance. When gate voltage is low, on-state resistance is relatively high, which will result in large on-state loss. After reaching a certain value, on-state resistance can be considerably reduced. Hence, a higher gate voltage is generally applied. In addition, gate voltage affects the switching characteristics of the device. The device switches faster with the increase in gate voltage, but the voltage should not be excessively high in case of oscillation. Moreover, a high gate voltage can adversely affect the reliability of the gate oxide, thereby resulting in device failure.

A negative gate voltage is generally required to reliably turn off the SiC MOSFET. The higher the voltage, the faster the device can turn off. Similarly, a large negative voltage adversely affects the reliability of the gate oxide. Based on the preceding analysis and combined with the datasheet provided by manufacturer, gate voltage is recommended to be −5/18 V.

#### 3.2.2. Gate Resistance

Gate resistance must be considered in the design of the drive circuit. If the gate resistance is large, then oscillation during the switching process can be suppressed, but the turn-on and turn-off speed will be slow. This condition may increase the loss of the device. By contrast, if the gate resistance is small, then the switching speed accelerates, which easily leads to current and voltage oscillation. However, the loss of the device will decrease. According to the analysis of Li et al. [28] and after repeated testing, gate resistance is determined to be 10 Ω.

#### 3.2.3. Driver Chip

At present, the driver chips available for SiC MOSFETs on the market are mainly IXYS_609 of IXYS (Milpitas, CA, USA) and ACPL-W346 of Avago (San Jose, CA, USA). The drive capability of IXVYS_609 is relatively stronger, which can provide peak current of 9 A. However, it has no isolation function. The drive capability of ACPL_W346 is weaker than that of IXVYS_609. It can provide a peak current of 2 A and is integrated with an optocoupler isolation. The peak current of 2 A is sufficient for a single SiC device. The drive circuit can be more compact due to the integrated optocoupler isolation, and the volume of the SSCB can be reduced. Therefore, the ACPL_W346 chip is selected. Table 3 lists the main technical parameters of ACPL_W346. The specific design scheme of the driving circuit based on ACPL_W346 is described in [29].

### 3.3. Fault Detection Circuit

**Figure 3:**
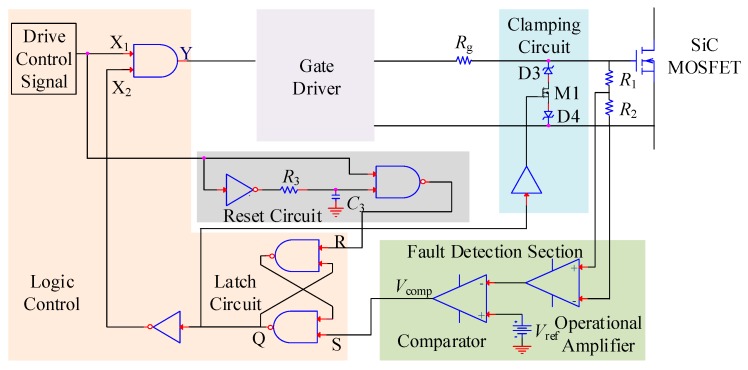
Schematic diagram of fault detection circuit.

During the fault process, the fault current will cause an evident change in the gate-source voltage because of the existence of the Miller capacitance [30]. Thus, the fault can be detected by the change of gate-source voltage. Figure 3 shows the schematic diagram of the fault detection circuit based on gate-source voltage variation.

**Figure 4:**
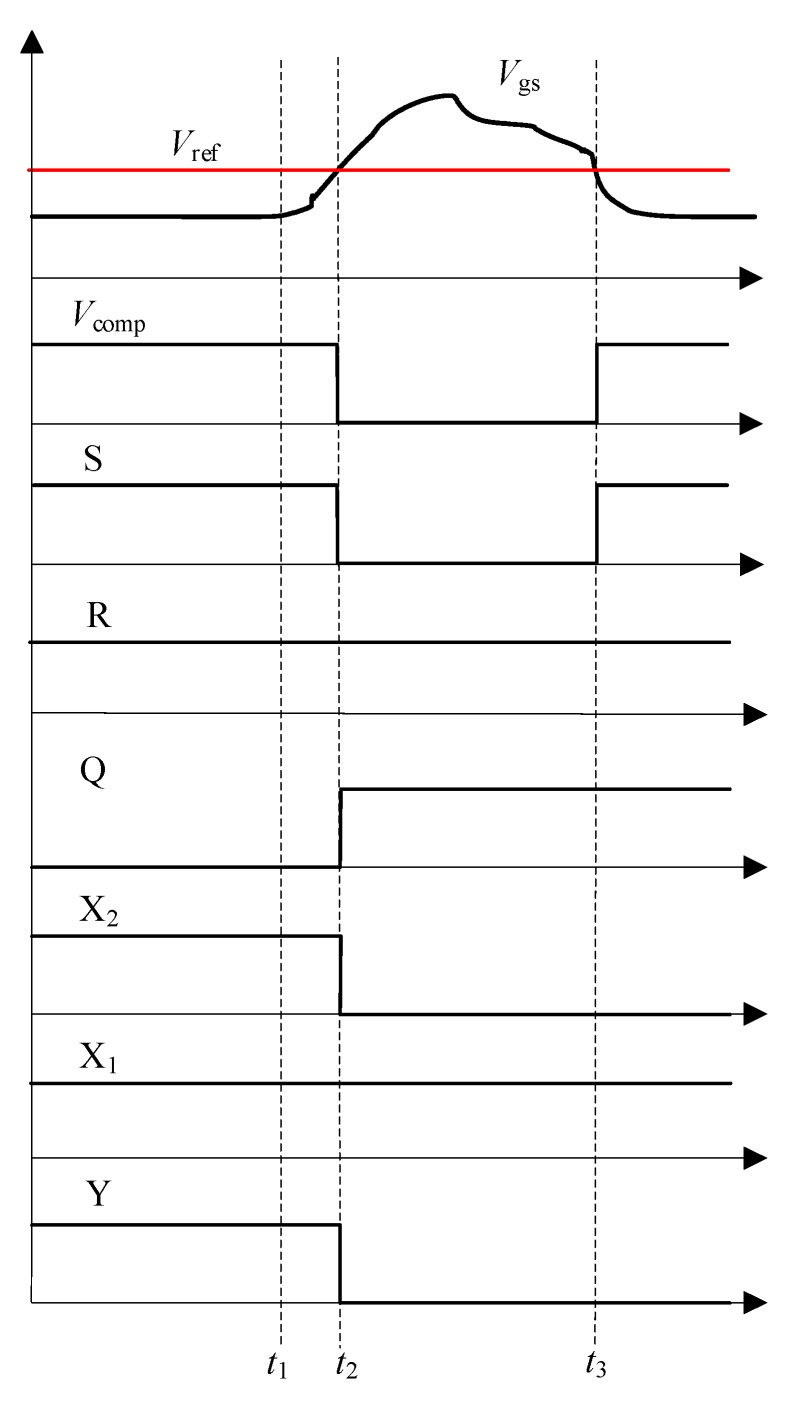
Sequence chart of fault detection circuit under fault condition.

As shown in Figure 3, the fault detection circuit is composed of clamping circuit, fault detection section, latch circuit, logic control circuit, and reset circuit. The clamping circuit is used to suppress the rise in gate voltage and avoid the overvoltage harm to the gate oxide. The divided gate-source voltage is sent to the differential operational amplifier, whose output is connected to the inverting input of the comparator, and the reference signal is connected to the non-inverting input. If the divided voltage exceeds the reference voltage, then the comparator output signal level switches from high to low. The SR latch circuit outputs a high level and remains. After sending the signal to the inverter, the signal logic reverses. The signal is then sent to the AND gate. During this time, even if the drive control signal is at a high level, the signal level of the AND gate output still remains low. This signal is the input signal of the drive circuit. In this way, the SSCB operates and the SiC MOSFET is turned-off. Figure 4 shows the sequence chart of the fault detection circuit under fault condition.

### 3.4. Design of the Energy Absorption Circuit

During the fault clearing period, the energy stored in the parasitic inductance of the circuit is dissipated by the energy absorption circuit, and the function is achieved by MOV_ex. The voltage on SSCB is clamped to *V*mov because of the clamping function of MOV. The voltage on inductance *L* can be expressed by:

(1) $$V_{L}=V_{\mathrm{MOV}}-V_{\mathrm{DC}},$$

where, *V*_DC_ is the DC-link voltage. To simplify the analysis, the change rate of fault current is assumed to be linear based on the simulation and test results in [31,32,33]. The inductance current, namely the current of SSCB, can then be calculated as follows:

(2) $$i_{L}=I_{\max}-\frac{V_{\mathrm{MOV}}-V_{\mathrm{DC}}}{L}t,$$

where *I*_max_ is the peak current. When SSCB starts to operate, the current will gradually attenuate to zero from its peak value. By setting *i*_L_ = 0, the total working time of SSCB *t*_s_ can be obtained.

(3) $$t_{s}=\frac{I_{\max}L}{V_{\mathrm{MOV}}-V_{\mathrm{DC}}}$$

The absorbed energy in the energy absorption circuit can then be achieved by:

(4) $$\begin{array}{cc} W_{\mathrm{MOV}\_\mathrm{ex}} & =\int_{0}^{t_{s}}V_{\mathrm{MOV}}\cdot i_{L}dt\\ & =\int_{0}^{t_{s}}V_{\mathrm{MOV}}\cdot (I_{\max}-\frac{V_{\mathrm{MOV}}-V_{\mathrm{DC}}}{L}t)dt\\ & =\frac{1}{2}(\frac{V_{\mathrm{DC}}}{V_{\mathrm{MOV}}-V_{\mathrm{DC}}})LI_{\max}^{2} \end{array}.$$

Since the inductance in the circuit is low (µH level), the energy that needs to be absorbed is small although the short circuit current is high. Thus, an ordinary MOV can meet the requirement of the design. In this study, *V*_DC_ is 400 V. After looking up the product model, 14D511K is selected as the MOV_ex. Main parameters of 14D511K are listed in Table 4.

### 3.5. Design of the Snubber Circuit

**Figure 5:**
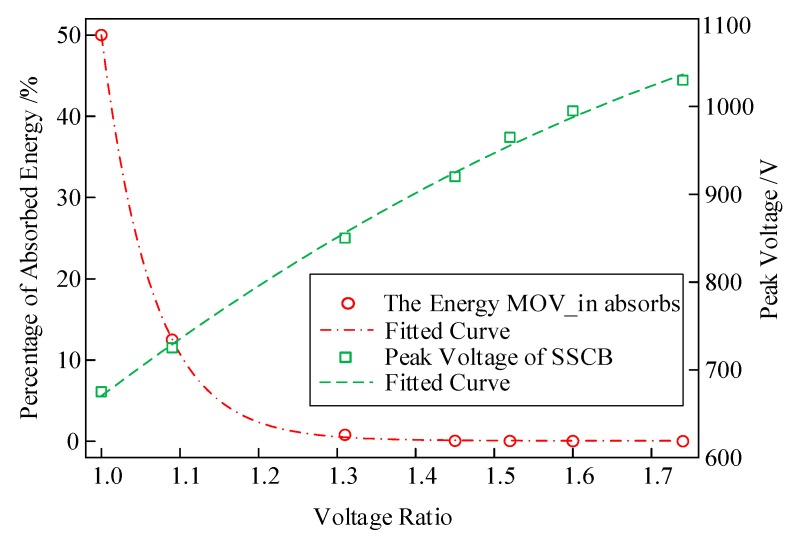
Absorbed energy and peak voltage at varying voltage ratio.

To suppress the voltage spike, MOV_in is used in the snubber circuit. The fault current will flow into MOV_in first due to smaller parasitic resistance in the snubber circuit. Then, the current will quickly transfer to MOV_ex and MOV_ex will continue to absorb the energy. Thus, MOV_in only works during the early turn-off stage and absorbs minimal energy. However, the voltage of MOV_in affects the peak voltage of SSCB, and the selection of MOV_in is related to MOV_ex. Figure 5 illustrates the simulated relationship of absorbed energy and peak voltage to voltage ratio. With the increase of breakdown voltage ratio of MOV_in and MOV_ex, MOV_in absorbs less energy, while the voltage overshoot of SSCB increases. When voltage ratio is greater than 1.3, the snubber circuit absorbs minimal energy and barely changes. Considering that the energy MOV_in absorbs and the peak voltage can be suppressed effectively, the breakdown voltage of MOV_in can be 1.3 times that of MOV_ex. Since the breakdown voltage of MOV_ex is selected to be 510V, 05D681K with the breakdown voltage of 680V is chosen. The main parameters are provided in Table 5.

## 4. Testing on the SSCB Prototype with SiC MOSFET

**Figure 6:**
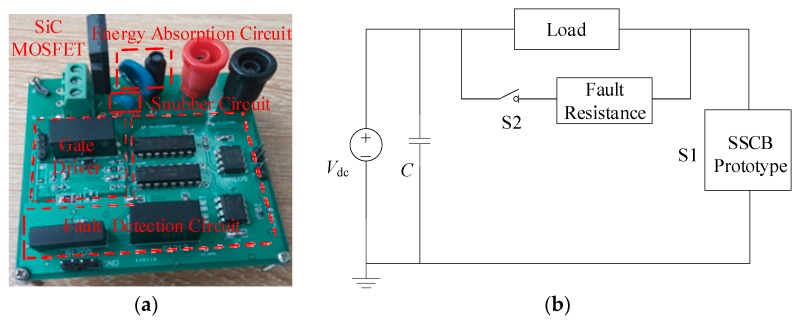
(**a**) SSCB prototype with SiC MOSFET and (**b**) fault simulation circuit.

**Figure 7:**
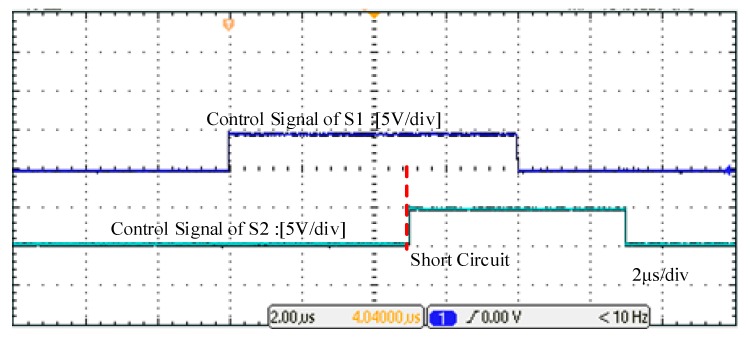
Sequence of control signals of S1 and S2.

Based on the structure of Figure 1b, the prototype of the SSCB with SiC MOSFET was developed and is shown in Figure 6a. The SSCB is composed of a SiC MOSFET, a gate driver, a fault detection circuit, an energy absorption circuit, and a snubber circuit. Gate driver uses the ACPL-W346 driver chip. Fault detection circuit is based on the schematic diagram in Figure 3. Energy absorption circuit is composed of MOV (14D511K) to absorb the energy. Snubber circuit is composed of MOV (05D681K) to suppress the voltage spike in the early turn-off period. The fault simulation circuit is shown in Figure 6b, where S1 is the SSCB prototype. Switch S2, which is parallel with the load, is another SiC MOSFET that controls on and off of the circuit. Load is represented by a 100 Ω resistance. Voltage-stabilizing capacitor *C* is 560 μF, and DC-link voltage is 400 V. First, S1 is on while S2 is off. The system is under normal working condition. After 5 μs, a turn-on signal is sent to S2, placing the system in a short circuit state, and the current rapidly increases. To prevent the abnormal state caused by the malfunction of SSCB, S1 is forcibly turned off after 3 μs of short circuit. The sequence of the control signals is shown in Figure 7.

**Figure 8:**
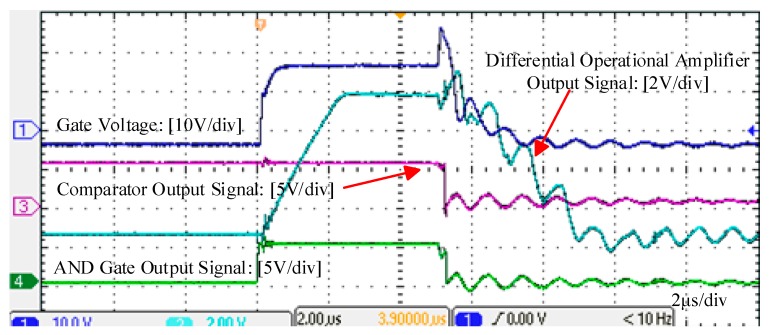
Waveforms of gate voltage change and test results of the fault detection circuit.

Figure 8 presents the test results of the fault detection circuit. When short circuit happens, gate voltage evidently rises. The differential operational amplifier can detect the change and react immediately. A turn-off signal is sent to the gate driver subsequently.

**Figure 9:**
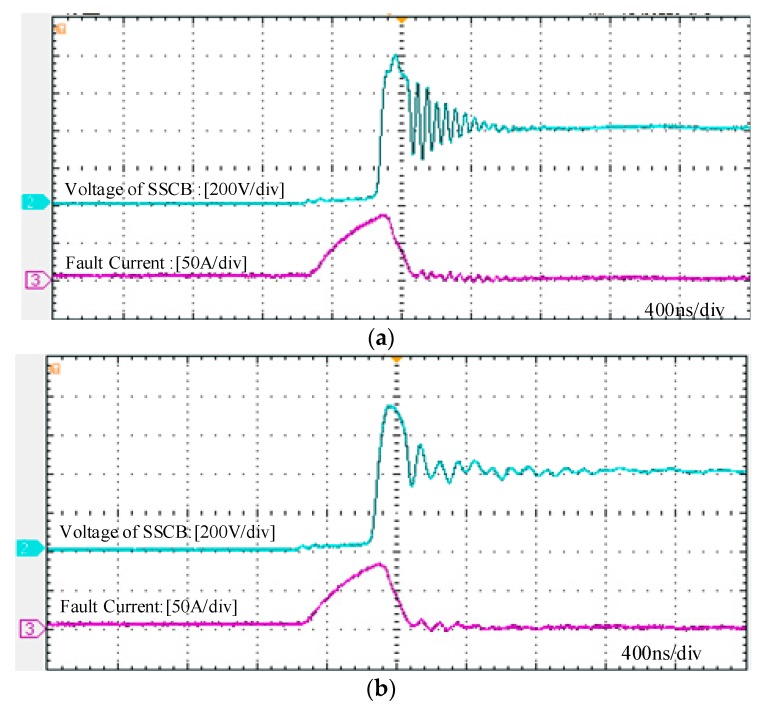
Waveforms of the fault current and voltage of the SSCB with SiC MOSFET: (**a**) without a snubber circuit and (**b**) with a snubber circuit.

Figure 9 shows the test results of the SSCB under the fault. The purple curve is the current waveform, whereas the blue curve is the voltage waveform. Figure 9a,b illustrate the cases where the snubber circuit is disconnected and connected to the circuit, respectively. When the snubber circuit is not connected, the peak voltage of SSCB during short circuit is 800 V, and oscillation is acute. Voltage spike can be suppressed to 750 V and the oscillation can be greatly restrained due to the existence of the snubber circuit. In addition, the peak fault current is approximately 80 A and the fault clearing time is about 720 ns. Therefore, the SSCB with SiC MOSFET can interrupt the current rapidly, and prevent the device from being exposed to high energy, long time shock, which provides protection for the device and improves the reliability of the system.

**Figure 10:**
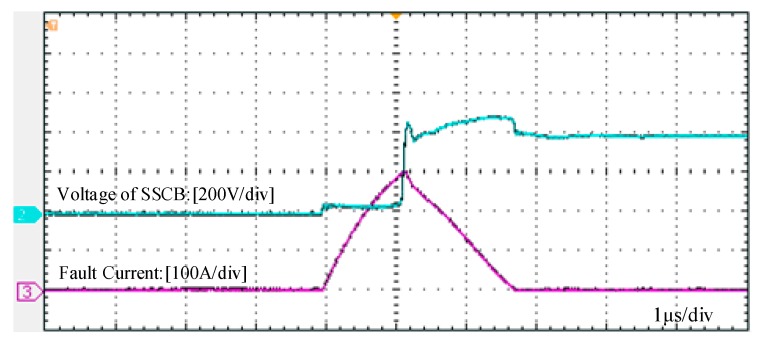
Waveforms of fault current and voltage of the SSCB with an Si insulated gate bipolar transistor (IGBT).

This study also does a test on an SSCB with Si IGBT, whose driver chip is TX-DA962D6 by LMY electronics, integrated with the desaturation detection function. Figure 10 shows the experimental result. The purple and blue curves represent the current waveform and voltage waveform, respectively. As shown in the figure, the fault clearing time is 2.8 μs and the fault current is up to 300 A. Both of these values are nearly four-fold those of the SSCB with SiC MOSFET. Such high current with such long time will exert huge electro-thermal stress on device and equipment in the system. 

In summary, by using an SiC MOSFET as the switching device, the designed SSCB prototype is evidently faster than SSCB with Si IGBT, which can cut off the fault current within 1 μs. The proposed SSCB with SiC MOSFET is distinguished by the fault detection circuit and the simplicity of the snubber circuit. On one hand, the fault detection method used in an Si-based SSCB is extended to an SSCB with SiC MOSFET because of the existence of a Miller capacitor in the SiC MOSFET. The fault can be detected rapidly and effectively based on the variation in gate voltage. On the other hand, by replacing the RC or RCD snubber circuit with MOV, the complicated design of parameters can be avoided and voltage spike can be suppressed as well. The detailed design in this study makes the realization of SSCB with SiC MOSFET easier and less complicated, providing reference for design and improvement of SSCB.

## 5. Conclusions

In order to realize the fast and reliable fault isolation in DC microgrid, the topology and structure of a 400 V miniature DC SSCB with SiC MOSFET are introduced. The design of the SSCB prototype is described in detail, including the selection of the device, drive circuit, current detection circuit, energy absorption circuit, and snubber circuit. The experimental results demonstrate that the SSCB with SiC MOSFET can immediately detect and interrupt the fault within 720 ns, peak current value of 80 A. Compared with the SSCB with Si IGBT, the proposed SSCB evidently has shorter interruption time and causes less thermal stress on the device.

## Figures and Tables

**Table 1:** Material properties of Si and SiC.

Parameter	Si	SiC
Bandgap/eV	1.1	3.2
Thermal Conductivity/(W/(cm·°C))	1.3	4.6
Electron Velocity/(10^7^ cm/s)	1	3

**Table 2:** Comparison of the 1200 V/80 mΩ CREE and ROHM SiC MOSFET.

Device	Temperature = 25 °C, *V*_DC_ = 600 V		Temperature = 200 °C, *V*_DC_ = 400 V	
	SCWT/μs	Critical Energy/J	SCWT/μs	Critical Energy/J
CREE	3.5	1.2	4.7	1.3
ROHM	4.7	1.6	5	1.4

**Table 3:** Main technical parameters of ACPL-W346.

Parameter	Minimal Value	Maximal Value
Source *V*_CC_/V	10	20
Output Current *I*_OUT_/A	-	2.5
Working Temperature *T*/°C	−40	105
Propagation Delay/ns	-	120
Common Mode Restraining Capability/kV·μs^−1^	50	-

**Table 4:** Main parameters of 14D511K.

Allowable DC Operating Voltage	Breakdown Voltage	Maximal Clamping Voltage	Maximal Absorbed Energy	Power
415 V	510 V	845 V	125 J	0.6 W

**Table 5:** Main parameters of 05D681K.

Allowable DC Operating Voltage	Breakdown Voltage	Maximal Clamping Voltage	Maximal Absorbed Energy	Power
560 V	680 V	1120 V	21 J	0.1 W
